# Efficient short read mapping to a pangenome that is represented by a graph of ED strings

**DOI:** 10.1093/bioinformatics/btad320

**Published:** 2023-05-12

**Authors:** Thomas Büchler, Jannik Olbrich, Enno Ohlebusch

**Affiliations:** Institute of Theoretical Computer Science, Ulm University, 89075 Ulm, Germany; Institute of Theoretical Computer Science, Ulm University, 89075 Ulm, Germany; Institute of Theoretical Computer Science, Ulm University, 89075 Ulm, Germany

## Abstract

**Motivation:**

A pangenome represents many diverse genome sequences of the same species. In order to cope with small variations as well as structural variations, recent research focused on the development of graph-based models of pangenomes. Mapping is the process of finding the original location of a DNA read in a reference sequence, typically a genome. Using a pangenome instead of a (linear) reference genome can, e.g. reduce mapping bias, the tendency to incorrectly map sequences that differ from the reference genome. Mapping reads to a graph, however, is more complex and needs more resources than mapping to a reference genome. Reducing the complexity of the graph by encoding simple variations like SNPs in a simple way can accelerate read mapping and reduce the memory requirements at the same time.

**Results:**

We introduce graphs based on elastic-degenerate strings (ED strings, EDS) and the linearized form of these EDS graphs as a new representation for pangenomes. In this representation, small variations are encoded directly in the sequence. Structural variations are encoded in a graph structure. This reduces the size of the representation in comparison to sequence graphs. In the linearized form, mapping techniques that are known from ordinary strings can be applied with appropriate adjustments. Since most variations are expressed directly in the sequence, the mapping process rarely has to take edges of the EDS graph into account. We developed a prototypical software tool GED-MAP that uses this representation together with a minimizer index to map short reads to the pangenome. Our experiments show that the new method works on a whole human genome scale, taking structural variants properly into account. The advantage of GED-MAP, compared with other pangenomic short read mappers, is that the new representation allows for a simple indexing method. This makes GED-MAP fast and memory efficient.

**Availability and implementation:**

Sources are available at: https://github.com/thomas-buechler-ulm/gedmap.

## 1 Introduction

In genomic studies and diagnostics, a single linear reference is commonly used to represent the human genome. However, there are a lot of differences between the genomes of individuals of the same species and a single reference is unable to cover them. The 1000 Genomes Project Consortium (2015) produced a database that includes the genomes of 2504 different humans and the differences between them. Including common variations in the reference gives a more accurate representation of the genomes of a species. Such a representation is called a pangenome. In most cases, a pangenome is constructed from a reference sequence and a VCF-file containing the variations. Roughly speaking, a pangenome can be seen as the set of all genomes of a species. Since the genomes share many identical substrings, they can collectively be represented by a sequence graph (The Computational Pan-Genomics Consortium 2016) or an elastic degenerate string (EDS) (Iliopoulos et al. 2017). Using a pangenome as the ground truth of scientific studies reduces reference allele bias since a common variation in a genome can be recognized as such and will not be treated as an error. Widely used next-generation sequencers produce short reads of a given DNA sample. A read is a substring of the sample that can include errors, and read mapping is the task of finding the locus in the genome the read emerged from. In read mapping, the reference bias has the consequence that reads may not be mapped correctly, e.g. if the allele of the sample differs from the reference allele. It has been shown (Garrison et al. 2018, Kim et al. 2019, Sirén et al. 2021) that the usage of a pangenome instead of a single reference sequence can cope with this problem. In the following, we briefly discuss this related work. HISAT2 (Kim et al. 2019) first builds a pangenome graph “by creating a linear graph from the reference genome and then adds mutations, deletions and insertions as alternative paths through the graph” (usually the variants are collected in a VCF file). HISAT2 “can incorporate insertions up to 20 base pairs (bp) and deletions of any length.” Thus, it does not incorporate complex structural variants, such as duplications, inversions or copy number variations. In a second step, HISAT2 constructs a genome graph index structure based on a method developed by Siren et al. (Sirén et al. 2014). In addition to the global index for representing the human genome plus a large collection of variants (the HISAT2 index covers 92% of known variants of the NA12878 genome), HISAT2 uses thousands of small indexes, each spanning ∼57 kb, which collectively cover the reference genome and its variants. This improves speed and accuracy. The VG-MAP algorithm (Garrison et al. 2018) incorporated complex structural variants into the pangenome graph, resulting in a complex sequence graph that contains cycles produced by duplications and complex genomic rearrangements. In the article that introduces Giraffe, the successor of VG-MAP, Sirén et al. (2021) write: “However, VG-MAP is at least an order of magnitude slower than popular linear genome mappers that have comparable accuracy. Given that mapping is frequently a bottleneck in genome analysis, the cost of VG-MAP has proven prohibitive.” For this reason, Giraffe does not incorporate structural variants other than large indels into its pangenome graph. Giraffe and HISAT2 both use a graph Burrows–Wheeler transform (GBWT) index, which is complex and difficult to understand. To speed up the mapping, Giraffe further takes reference haplotypes into account (paths in the pangenome graph that are observed in individuals’ genomes).

In this article, we define a new representation of pangenomes. Our representation encodes single nucleotide polymorphisms (SNPs) as well as small insertions and deletions (indels) linearly and in place using a method similar to ED strings and the vBWT (Maciuca et al. 2016), but with a constant alphabet size. Large indels and copy number variations are represented in a graph structure. Among the advantages of our encoding is good readability and the availability of a simple coordinate system. This means that humans can directly see small variants in the sequence, without the need of plotting a graph.

Moreover, we show how our representation can be used for short read mapping. The problem of matching (i.e. mapping without errors) patterns to ED strings without indexing is well studied (Grossi et al. 2017, Aoyama et al. 2018, Cisłak et al. 2018, Procházka et al. 2021). However, our approach allows errors and uses an index to accelerate the process. Therefore, it is more comparable to pangenomic short read mappers. The coordinate system resulting from our encoding enables us to directly apply position-based indexes, e.g. the minimizer index. We show how common methods like calculating a minimizer index, finding good seeds with the help of the index and aligning using dynamic programming can be adjusted to work efficiently on our representation. We compare our software tool GED-MAP with state-of-the-art tools for short read mapping to pangenomes on simulated and real datasets at a whole human genome scale. While the differences in the mapping accuracy are neglectable (except for structural variants), there are significant differences in the resources required to construct the index as well as in the mapping process. The experiments show that GED-MAP is fast and memory efficient because the new representation of pangenomes allows for a rather simple indexing method.

## 2 Materials and methods

### 2.1 Preliminaries

In the following, Σ denotes the alphabet of bases, i.e. Σ={A,C,G,T}. For a string *S*, we denote its length by |S|. Furthermore, *S*[*i*, *j*] is the substring of *S* that starts at position *i* and ends at position *j*. In this article we use 1-based indexing. The following definitions specify *Elastic-Degenerate strings (EDS)*.

Definition 1 (Elastic-Degenerate symbol). An *Elastic-Degenerate symbol (ED symbol)* $\hat{u}$ over Σ is a nonempty and finite set of strings over Σ, i.e. $\hat{u}=\{u_{1},\ldots ,u_{m}\}$ with $u_{i}\in \Sigma^{*}$ for 1≤i≤m. A string $u_{i}\in \hat{u}$ is called an alternative. We will use the notation $\left(u_{1}|\ldots |u_{m}\right)$ for $\hat{u}$ (and simply write $u_{1}$ if m=1). Furthermore, the special symbol N stands for (A|C|T|G).

Definition 2 (Elastic-Degenerate string). An *Elastic-Degenerate string (EDS)* $\hat{S}$ over Σ is a sequence of ED symbols, i.e. $\hat{S}={\hat{u}}_{1}\ldots {\hat{u}}_{n}$.

Definition 3 (Matching). A string $S\in \Sigma^{*}$ matches an EDS $\hat{S}={\hat{u}}_{1}\ldots {\hat{u}}_{n}$ if and only if there are $u_{1},\ldots ,u_{n}$ with $u_{i}\in {\hat{u}}_{i}$ (for 1≤i≤n) such that $S=u_{1}\ldots u_{n}$.

Definition 4 (Occurrence). Let $P\in \Sigma^{*}$ be a pattern and $\hat{S}={\hat{u}}_{1}\ldots {\hat{u}}_{n}$ be an EDS. Let *p* be a position in $\hat{S}$ belonging to the alternative $u\in {\hat{u}}_{i}$ (1≤i≤n) and let $u^{\prime }$ be the suffix of *u* starting at this position. We say *P* occurs at position *p* in $\hat{S}$, if *P* matches a prefix of $u^{\prime }{\hat{u}}_{i+1}\ldots {\hat{u}}_{n}$.

In our notation (resembling regular expressions), an ED string is a string over the alphabet Σ∪{N,(,|,)}. For instance, $\hat{S}=$NG(A|)CA(AT|TA)GA is an EDS. Here, (A|) is an optional A and (AT|TA) is an ED symbol with the alternatives AT and TA. The length of $\hat{S}$ equals its length as ordinary string (here $|\hat{S}|=17$). The strings AGACATAGA and GGCAATGA are matching $\hat{S}$ and the patterns GACA and GCAT both occur at position 2 of $\hat{S}$, and ATGA occurs at position 10 of $\hat{S}$.

Note that our definition of occurrence is more specific than the standard definition in the literature (Iliopoulos et al. 2017). Usually, the position of an occurrence refers only to the index of the ED symbol. So, in the example ATGA would occur at position 4 because it starts in (AT|TA), which is the fourth ED symbol. However, this does not provide any information about the position inside the alternative. The alternatives in an ED symbol can be very long in real-world data and therefore we want to be able to address bases inside an alternative. In our definition, the occurrence refers to the coordinate (see Section 2.3) of the first base of the pattern.

**Figure 1.**
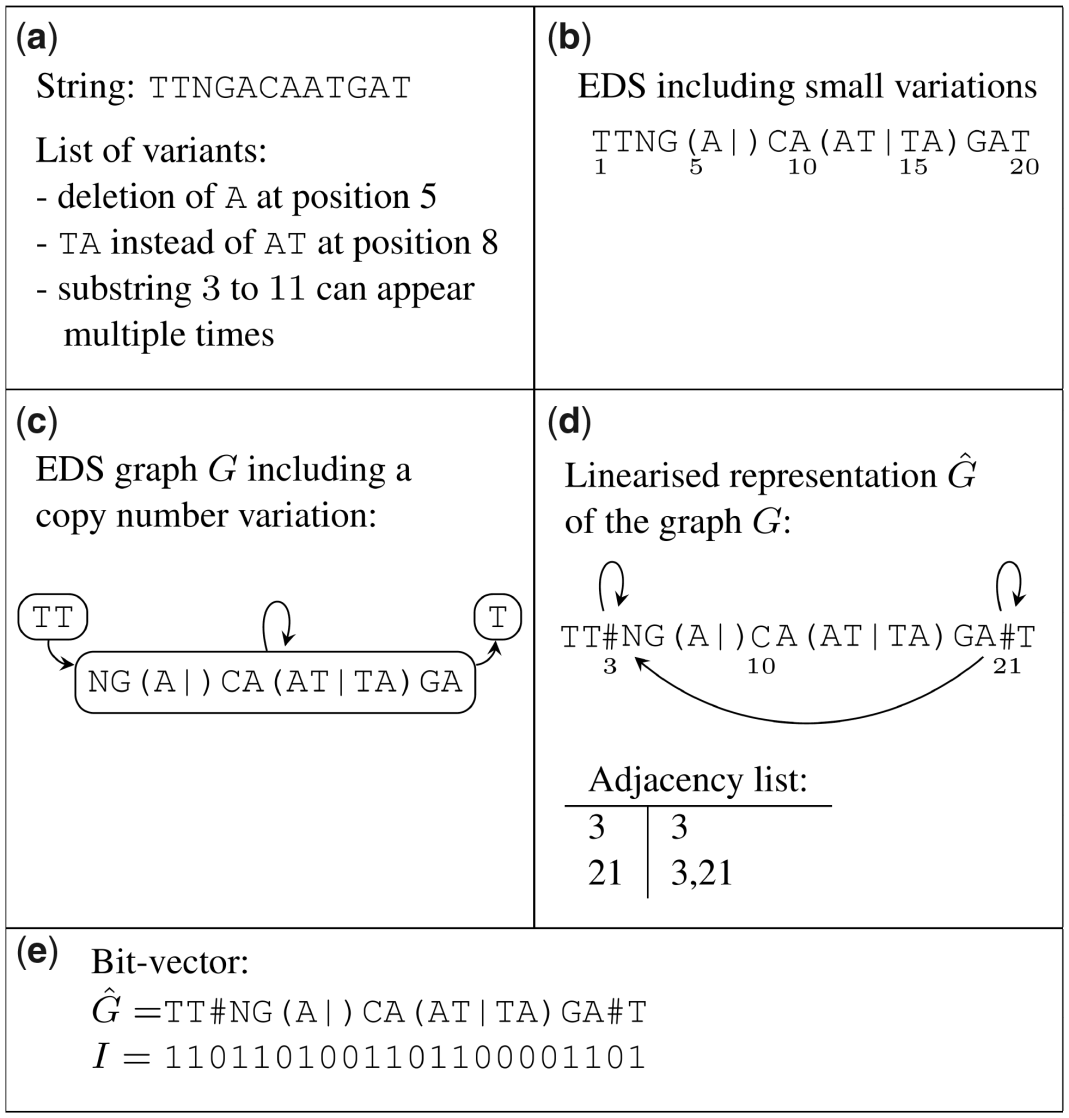
The figure shows the transformation from (a) a linear reference and a list of variants to (b) an EDS, (c) an EDS graph, and (d) its linearized form. In (e) one can see the additional bit-vector *I*, which indicates whether or not a symbol originates from the linear reference.

We generate the EDS from a FASTA file containing a reference sequence and a VCF-file containing the variations. Similar to other tools, we ignore overlapping variants in the VCF-file. Figure 1a and b shows the transition from a sequence and a list of variations to an EDS. The copy number variation is not represented in the EDS but in the EDS graph, which we will define next.

### 2.2 Pangenome as EDS graph

An EDS, as defined above, can represent simple genomic variations like SNPs and indels, but not complex or cyclic variations. We will represent variations that only affect one or a few bases like SNPs and small indels in the EDS syntax. To properly represent structural variations like copy number variations and large indels, we use a directed graph G=(V,E).

Each node v∈V corresponds to an EDS l(v), we say *v* is *labeled* with the EDS l(v). The nodes and edges of *G* are formed by adding structural variations to the EDS $\hat{S}$. For example, if the substring $\hat{S}[i,j]$ can appear multiple times, we split $\hat{S}$ into three nodes with the labels $\hat{S}[1,i-1]$, $\hat{S}[i,j]$, and $\hat{S}[j+1,|\hat{S}|]$. (Since we want the three new labels to be syntactically correct ED strings, the positions *i* and *j* cannot be inside parenthesized ED symbols.) We connect them by directed edges in the order of appearance and add a self-loop to the middle node. Such a graph is depicted in Fig. 1c. We say a string *S* matches the EDS graph *G* if and only if there is a walk $v_{1},\ldots ,v_{m}$ such that $v_{1}$ is a source node in *G*, $v_{m}$ is a sink node in *G*, and *S* matches the EDS $l(v_{1})\ldots l(v_{m})$. Note that every EDS graph can easily be transformed into an equivalent sequence graph. In a sequence graph, the nodes are associated with ordinary strings or even single characters. (To construct the sequence graph from an EDS graph, one has to add a new node for each alternative in each degenerate symbol $\hat{u}$ and connect this node to degenerate symbols before and after $\hat{u}$.) The variation graph, the fundamental data structure of VG, is a sequence graph enhanced with additional information. However, the sequence graph requires more nodes and edges than our representation and therefore more memory.

We store the node labels of an EDS graph *G* as a string $\hat{G}$, where $\hat{G}$ is the concatenation of all node labels, separated by a special separator symbol #. The node labels are concatenated in the order of their appearance in the linear reference, which enables us to easily transform the coordinates in $\hat{G}$ into coordinates in the linear reference (see Section 2.3). Suppose that separators in $\hat{G}$ are numbered consecutively, starting with 1. Let sep(v) be the number of the separator immediately before the EDS l(v) of node *v* of *G*, where sep(v)=0 if there is none. For an edge $\left(v,v^{\prime }\right)$ in *G*, we add an edge from sep(v)+1 to $\mathrm{sep}(v^{\prime })$ to $\hat{G}$. These edges are stored in an adjacency list. When reading the sequence $\hat{G}$ in forward direction (from left to right), these new edges, or jumps (Büchler and Ohlebusch 2020), are the connections between all possible adjacent bases. In Fig. 1d for instance, when the separator at position 21 is encountered, the following bases are the ones behind positions 3 and 21. (Later we have to traverse the sequence in backward direction as well. Therefore, we additionally store the inverted adjacency list.) From now on, we identify an EDS graph with its representation $\hat{G}$ and use the example in Fig. 1 for all our explanations.

### 2.3 Coordinate system

A *coordinate system* is a system that uses coordinates to uniquely address bases in the reference (pan)genome. In a linear reference the coordinate is just the offset. A base can, e.g. be addressed as chromosome 2 with offset 800 000. A *sensible* coordinate system should meet several requirements. It should be *readable*, *monotone*, and *spatial* (The Computational Pan-Genomics Consortium 2016, Rand et al. 2017). In other words, the coordinates should be concise and comprehensible, increase along the genome and nearby bases should have similar coordinates.

Since the EDS graph is stored as a string $\hat{G}$ (plus adjacency lists), it naturally provides a concise coordinate system: the coordinate of a base is its position in the string. This coordinate system meets the aforementioned criteria. The coordinate of a base in $\hat{G}$ is its offset from the start of $\hat{G}$. In the absence of variants, it coincides with the coordinate in the linear reference.

On monotonicity: Let *S* be a string of length *n* that matches $\hat{G}$ without cycles and further let c(S[i]) be the coordinate of base *S*[*i*] in $\hat{G}$, for 1≤i≤n. Then it is true that c(S[i])<c(S[i+1]), for 1≤i<n, and hence the coordinates are monotone. For example, S=TTAGACATAGAT matches the graph $\hat{G}$ in Fig. 1d and the sequence of the coordinates $\left(c(S_{1}),\ldots ,c(S_{\left|S\right|})\right)$ is (1,2,4,5,7,10,11,16,17,19,20,22). As one can see, this sequence is monotone. If *S* matches $\hat{G}$ with cycles, the coordinates cannot per se be monotone.

On spatiality: When there are no variants between two coordinates, the difference between the coordinates is the distance between bases. When there are variants of total length *x* between two coordinates, the difference between the coordinates is roughly the distance between the corresponding bases plus *x*. If there are mostly short variations in the EDS, *x* tends to be small.

Since the coordinates in the linear reference may still play an important role, we show how to transform a coordinate in $\hat{G}$ to a coordinate in the linear reference. This transformation uses an additional bit-vector *I* and rank queries on *I*. After a linear preprocessing and with the help of an additional data structure, the rank value can be calculated in constant time (Jacobson 1989). The bit-vector *I* indicates whether the symbol at position *i* in $\hat{G}$ originated from the linear reference sequence or not. If not, the symbol is a syntax symbol or part of an alternative sequence. If a base $\hat{G}[p]$ originates from the linear reference, then its position in the linear reference is rank(I,p), where rank(I,p) is the number of ones in the vector *I* up to position *p*. In Fig. 1e, the vector *I* has ones at the positions of the bases AT in the second variant, because AT stems from the linear reference, whereas *I* has zeros at the positions of TA. The base G right behind the last bracket corresponds to the tenth one-bit in *I* and therefore originates from the G at the tenth position in the linear reference.

### 2.4 Minimizer index

A string over Σ of length *k* occurring in the pangenome (here EDS graph $\hat{G}$) is called *k-mer*, and a *k-mer index* provides efficient access to the positions of all occurrences for each k-mer. In order to map a read to the pangenome, one can use a k-mer index to efficiently determine the positions of exactly matching k-mers, which subsequently can be used to find alignments.

Without the usage of minimizers, a k-mer index for the whole human genome requires tens of gigabytes of memory and actually contains more information than we need for the task of short read mapping. To reduce the amount of storage we use a *minimizer index* (Roberts et al. 2004) instead. A (w,k)*-minimizer* is the smallest among all k-mers starting in a window of size *w* in $\hat{G}$. As in Li (2016), we apply a hash function to the lexicographic rank of a k-mer and choose the k-mer with the smallest hash value as a minimizer. However, to keep the examples simple, we will use the smallest k-mer w.r.t. the lexicographic ordering as a minimizer in this article.

**Figure 2.**
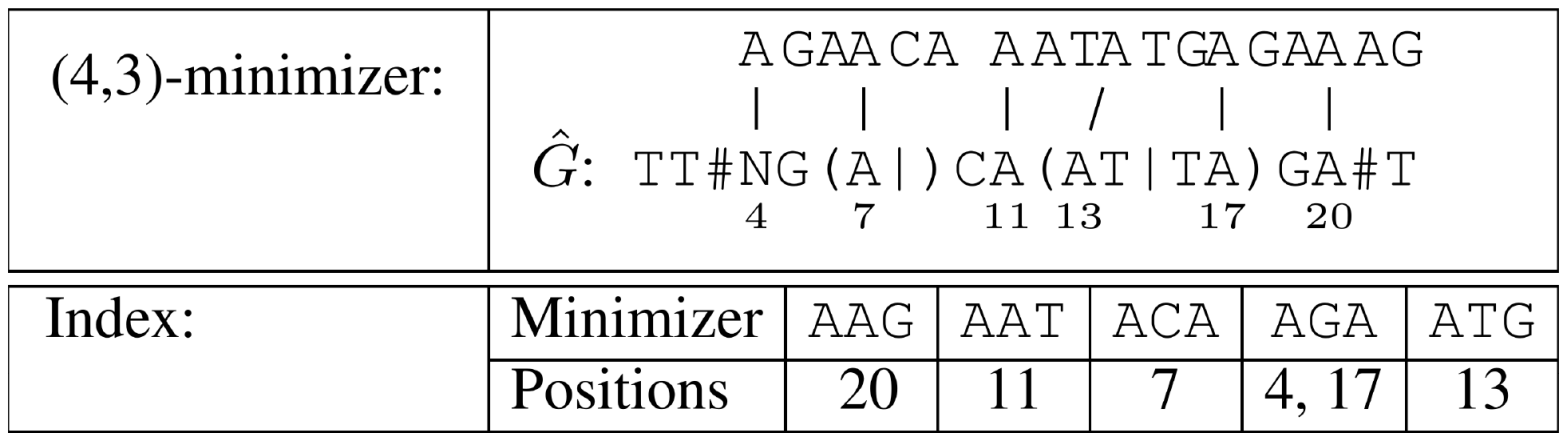
(4,3)-Minimizers of $\hat{G}$ and the index that maps the minimizers to their positions.

For example, we determine all (4,3)-minimizers of our example graph $\hat{G}=\mathrm{TT}\#\mathrm{NG}(\mathrm{A|})\mathrm{CA}(\mathrm{AT|TA})\mathrm{GA}\#T$ (see Fig. 1). The first (leftmost) window includes all 3-mers occurring at positions in the interval [1, 4]. The 3-mers occurring at position 1 are TTA, TTC, TTG, and TTT, at position 2 TAG, TCG, TGG, and TTG, and at position 4 AGA, CGA, GGA, TGA, AGC, CGC, GGC, and TGC. (In accordance with Def. 4, no k-mer occurs at a position with a syntax symbol.) The lexicographically smallest among all these 3-mers is AGA occurring at position 4, hence it is stored in the minimizer index. The same 3-mer is also the smallest one in the windows (intervals) [2, 5] and [3, 6]. In the interval [4, 7], the 3-mer ACA at position 7 is the smallest, thus it is stored in the minimizer index. The remaining minimizers are: ATG at position 13, AGA at position 17, and AAG at position 20. Figure 2 shows all (4,3)-minimizers of $\hat{G}$ and a table that maps the minimizers to their positions. This table represents the minimizer index. The layout of our minimizer index used in our implementation is discussed in the Supplementary Material.

*Construction of the minimizer index*: In contrast to standard strings, there can be multiple k-mers occurring at a position in an EDS graph $\hat{G}$. To compute the minimizers, we first compute the set of k-mers occurring at each position and for each position determine the smallest k-mer. Then we slide a window over these k-mers (which are sorted by their position) and iteratively mark the smallest k-mer per window. The k-mer index is then constructed from the marked k-mers.

In the following, we explain how to efficiently determine the set of k-mers occurring at each position. First, we ignore the graph structure and consider an arbitrary EDS $\hat{S}$. The set of k-mers occurring at a position *p* could be calculated by reading all possible paths in forward direction starting at *p*. A better approach is scanning $\hat{S}$ from right to left once and deducing the set of k-mers $K_{p}$ occurring at position *p* from $K_{p+1}$. The variations are accounted for with the help of two special sets $K_{r}$ and $K_{l}$. Table 1 depicts the example used in the following. In the simple case where $\hat{S}[p],\hat{S}[p+1]\in \Sigma$, the set $K_{p}$ is obtained by prepending $\hat{S}[p]$ to each k-mer in $K_{p+1}$ and deleting the last character of each resulting string. In the example CAA$\in K_{1}$ because $\hat{S}[1]=C$ and AAT$\in K_{2}$.

**Table 1.** Contents of the sets $K_{p}$, $K_{r}$, and $K_{l}$ while iterating over the EDS $\hat{S}=\mathrm{CA}(\mathrm{AT|TA})\mathrm{GA}$ with k=3.^a^

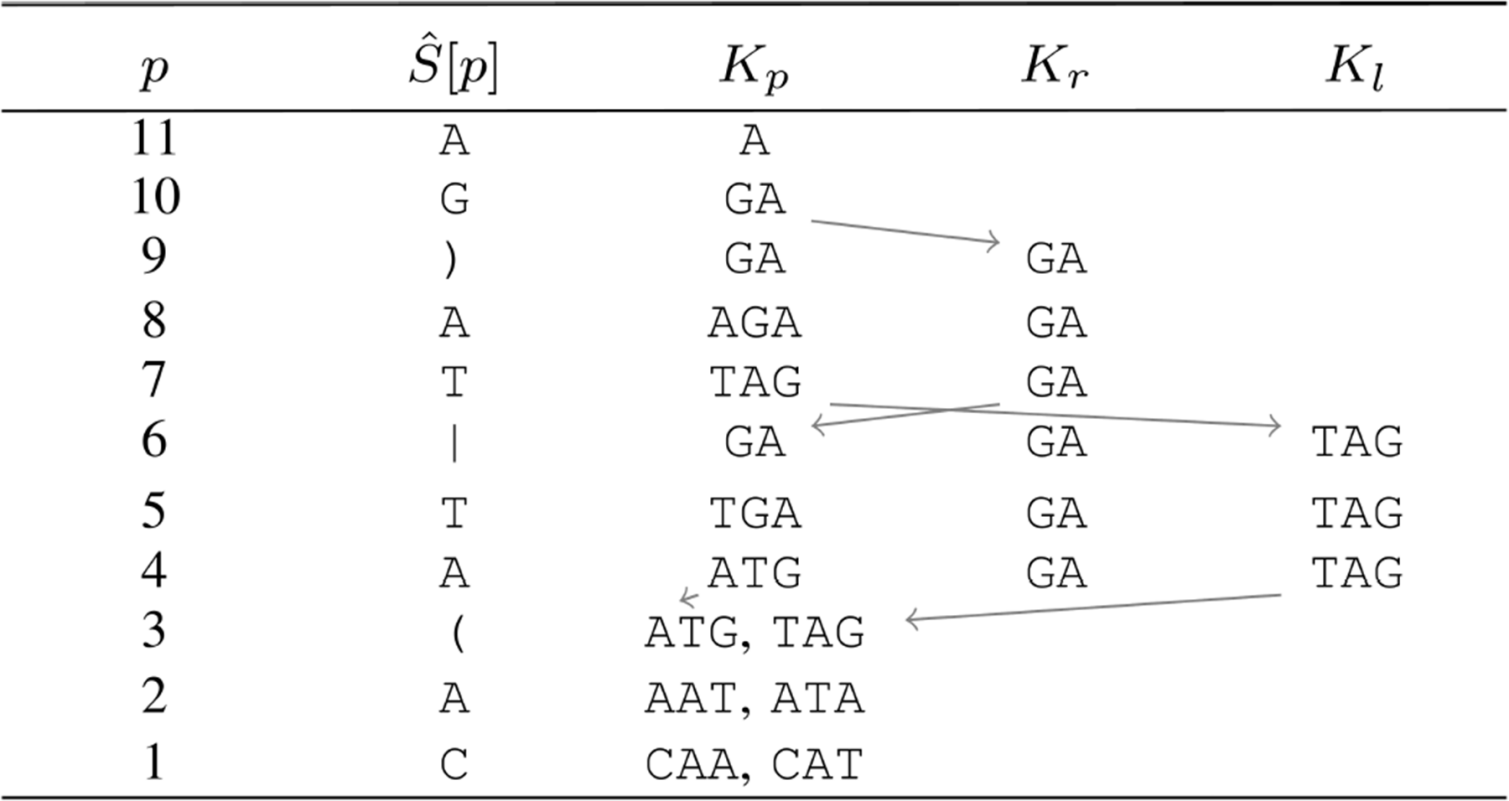

a Only at positions with syntax symbols (here at p=3,6, and 9) the sets $K_{r}$ and $K_{l}$ are used.

If $\hat{S}[p]\in \Sigma$ and $\hat{S}[p+1]\in \{),|\}$, $\hat{S}[p]$ is the last character in an alternative. By definition of an EDS, $K_{p}$ can be obtained in the same way from $\hat{S}[p]$ and $K_{r}$, where *r* is the right boundary of the current ED symbol. In our example $K_{5}$ and $K_{8}$ are obtained from $K_{10}$. Because we do not store the entire table, we set $K_{r}\leftarrow K_{10}$ when we see the bracket “)” at position 9. Then we (implicitly) set $K_{6}$ and $K_{9}$ to $K_{r}$. Now $K_{5}$ and $K_{8}$ can be calculated as in the simple case above. At the left boundary *l* of an ED symbol, $K_{l}$ is the union of all k-mer sets of the alternatives. This union is calculated step by step. Whenever we reach the end of an alternative, we add the elements of $K_{p}$ to $K_{l}$ (see line p=6 in the example). At the end of the last alternative, we add the elements of $K_{l}$ to $K_{p}$. This happens in line p=3 in the example.

Because we determine the minimum k-mer by a hash value and not its lexicographic rank, the minimum k-mer in $K_{p}$ cannot be obtained from the minimum k-mer of $K_{p+1}$. Furthermore, we treat the character N in a special way. While calculating the sets, we allow N in the k-mers (hence $K_{p}$, $K_{r}$, and $K_{l}$ are actually containing strings over the alphabet Σ∪{N}). When calculating the minimum of a set, we replace every N with each character in Σ and then calculate the hash value. We consider substrings that contain many N as not helpful and therefore ignore them.

To compute the minimizer index of an EDS graph $\hat{G}$, we first determine the minimum k-mer for each position in $\hat{G}$. Let *v* be such a node and let l(v) be its label. Remember that l(v) is an EDS. The only k-mers that are not considered to be minimizers are those that span over an edge in $\hat{G}$. For each node $v^{\prime }$, let $K[v^{\prime }]$ be the set of k-mers starting at the first position in $l(v^{\prime })$. For a node *v* we define $K_{|l(v)|+1}=\underset{(v,v^{\prime })\in E}{\cup }K[v^{\prime }]$, i.e. the set of k-mers starting behind l(v) as the union of all sets of k-mers starting at a successor of *v*. Then we proceed as in the algorithm above, starting at position |l(v)| in *v*. (When *k* characters have been added to all k-mers in $K_{|l(v)|+1}$, we can halt since from then on, the k-mers do not span over an edge and therefore were already calculated before.)

There can be minimizers that occur at an extraordinarily large number of positions. These positions are not very helpful in the mapping algorithm. Moreover, considering all of them will significantly slow down the mapping process. To cope with this problem, we trim the index by removing all minimizers from the index that occur at more positions than a given threshold. An evaluation on how this trimming affects the index size and the mapping can be found in the Supplementary Material.

### 2.5 Short read mapping

A read is a DNA sequence generated by a sequencer. Today’s most common sequencers are still using next-generation sequencing technologies and produce short reads with a length of up to a few hundred base pairs. Roughly speaking, read mapping is the task of finding the locus of such a read in the genome. A read may contain errors and variants that are not present in the reference pangenome, i.e. it may not match the reference exactly. Thus, one is interested in an optimal semi-global alignment between the read and the reference (i.e. the read is aligned with a substring of the reference). We solve this task by calculating the minimizers of a read, looking up their positions in the minimizer index, ranking the positions, and calculating a semi-global alignment of the read with the reference at the best-ranked positions. This process will be described in more detail below.

#### 2.5.1 Seed determination

Given a read *r*, we determine its minimizers and query their positions in $\hat{G}$ from the minimizer index. This results in a sequence of pairs of positions in $\hat{G}$ and positions in *r*. We then rank the pairs of this sequence and later use the top ranked pairs as seeds in the alignment procedure. Our ranking will prefer minimizers that occur close to other minimizers in $\hat{G}$ and is described in more detail below.

The idea of combining several exact matches (and a matching minimizer is an exact match) was first used as “two-hit” method in gapped BLAST (Altschul et al. 1997). Therein, “two-hit” refers to two k-mers on the same diagonal, i.e. two k-mers with the same difference between their position in the read and their position in the string. Because alternatives can have different lengths, we cannot directly apply this. Our method is more similar to the chaining used by MUMmer (Delcher et al. 1999).

Roughly speaking, we search for multiple minimizers of *r* that occur close to each other in $\hat{G}$. This is achieved by sorting the pairs by the corresponding position in $\hat{G}$ and then scanning the sorted list. Since the positions for one minimizer are already stored in a sorted list in the minimizer index, we can obtain a sorted list of the occurrences of all minimizers by iteratively merging sorted lists. Consider, e.g. the mapping of the read r=TTAGAATCGA to the EDS graph $\hat{G}$ from Fig. 1. The (4,3)-minimizers of *r* are AGA occurring at position 3, AAT at position 5, ATC at position 6, and CGA at position 9. The positions of these minimizers in $\hat{G}$ can be queried in the index (see Fig. 2). These positions are 4 and 17 for AGA, 11 for AAT, and no positions for ATC and CGA. So, we get the position pairs (3,4),(3,17), and (5,11).

To rank the pairs, we define the term *colinear*. Two pairs (i,j) and $\left(i^{\prime },j^{\prime }\right)$ are colinear if and only if $i<i^{\prime }$ and $j<j^{\prime }$. We denote this by $\left(i,j){<}_{c}(i^{\prime },j^{\prime }\right)$. A colinear chain of length ℓ is a sequence of pairs $\left(i_{1},j_{1}),(i_{2},j_{2}),\ldots ,(i_{l},j_{l}\right)$ with $\left(i_{q},j_{q}){<}_{c}(i_{q+1},j_{q+1}\right)$ for 1≤q<ℓ. The score of a pair (i,j) is the length of a maximum colinear chain ending at (i,j). In our example above, (3,4) and (3,17) have score 1 and (5,11) has score 2, since $\left(3,4){<}_{c}(5,11\right)$. Hence, (5,11) will be used as seed for the alignment algorithm. If this seed does not yield a satisfying alignment, we will try seeds with lower scores.

We additionally ensure that all positions in $\hat{G}$ in such a chain are in a certain range, i.e. $j_{\ell }-j_{1}<\lambda$ for a parameter λ. It is obvious that the positions in the read *r* are in a range of size |r|, because they are all ≤|r|. We choose λ to be greater than |r|, but in the same order of magnitude (e.g. λ=2|r|). This ensures that only minimizers that are close together in the genome will be chained.

Let the sorted list of pairs $\left(i_{1},j_{1}),\ldots ,(i_{m},j_{m}\right)$, with $j_{\ell }\leq j_{\ell +1}$ for 1≤ℓ<m, be given. For each ℓ we consider the largest $\ell^{\prime }\leq m$ with $j_{\ell^{\prime }}-j_{\ell }<\lambda$. The problem of calculating the scores of the pairs $\left(i_{\ell },j_{\ell }),\ldots ,(i_{\ell^{\prime }},j_{\ell^{\prime }}\right)$ then relates to the problem of finding a longest increasing subsequences and hence can be calculated in O(x log x) time, where $x=\ell^{\prime }-\ell$ (Fredman 1975).

#### 2.5.2 Aligning

After a seed $\left(i^{*},j^{*}\right)$ has been determined, we compute a semi-global alignment of the read and the EDS graph $\hat{G}$. The alignment is based on affine gap costs (the edit distance can be used as a special case of affine gap costs as well). To keep the presentation simple, we show how an alignment that minimizes the edit distance can be calculated. Semi-global alignment means that the whole read *r* is aligned with a substring of $\hat{G}$, enforcing that $r[i^{*}]$ is aligned with $\hat{G}[j^{*}]$. First, we explain the alignment w.r.t. an EDS $\hat{S}$. More specifically, we determine the smallest edit distance between *r* and $\hat{S}[lo,hi]$ among all *lo* and *hi* with $lo\leq j^{*}\leq hi$ under the assumption that in an optimal alignment $r[i^{*}]$ is aligned with $\hat{S}[j^{*}]$ (or determine that the edit distance must be greater than some parameter *d*).

This is done using a variant of the well-known dynamic programming algorithm for the edit distance. Note that we can independently align $r[1,i^{*}]$ and $r[i^{*},n]$ with $\hat{S}[1,j^{*}]$ and $\hat{S}[j^{*},|\hat{S}|]$, respectively. Hence, we only describe the alignment starting from $\left(i^{*},j^{*}\right)$ in forward direction; the backward direction works analogously and the combination of both gives the complete alignment. Furthermore, we impose an upper bound *d* on the error and stop if it becomes apparent that the edit distance must be larger than *d*. Note that an edit distance of $d^{\prime }\leq d$ in forward direction imposes an upper bound of $d-d^{\prime }$ onto the backwards alignment.

Let $D_{i,j}$ be the edit distance between $r[i^{*},i]$ and $\hat{S}[j^{*},j]$. The minimum number of errors in a semi-global alignment is then $\min_{j\geq j^{*}}D_{\left|r,j\right|}$. The optimal alignment itself can be found by tracing the optimal solution back through *D*.

Table 2 shows the recurrence relations adapted to EDS and Table 3 gives an example of the dynamic programming table *D*. The example is showing the alignment in forward direction of *r* with $\hat{G}$, using seed (5,11). The definition in Table 2 includes the standard definition (1a–1d) and cases to cope with alternatives (2–4). In the table *D*, if a column *j* belongs to the ED symbol $\hat{u}$ and $\hat{S}[j]\in \{$(,|}, then column *j* is defined to be a copy of the column immediately preceding $\hat{u}$, see cases (2) and (3). If $\hat{S}[j]=$ “)”, then *j* is the end of an ED symbol $\hat{u}$. In this case, the preceding columns of column j+1 are all columns $j^{\prime \prime }$ that represent the end of an alternative $u\in \hat{u}$. Since we define an entry $D_{i,j}$ in column *j* as the minimum of the entries $D_{i,j^{\prime \prime }}$ of all columns $j^{\prime \prime }$ that precede column j+1 (4), column j+1 can be calculated from column *j*. Instead of searching for the values of $j^{\prime }$ and $j^{\prime \prime }$ in cases 3 and 4, we can cache these values whenever case 2 or 3 occurs and empty the cache in case 4.

**Table 2.** Inductive definition of *D*.^a^

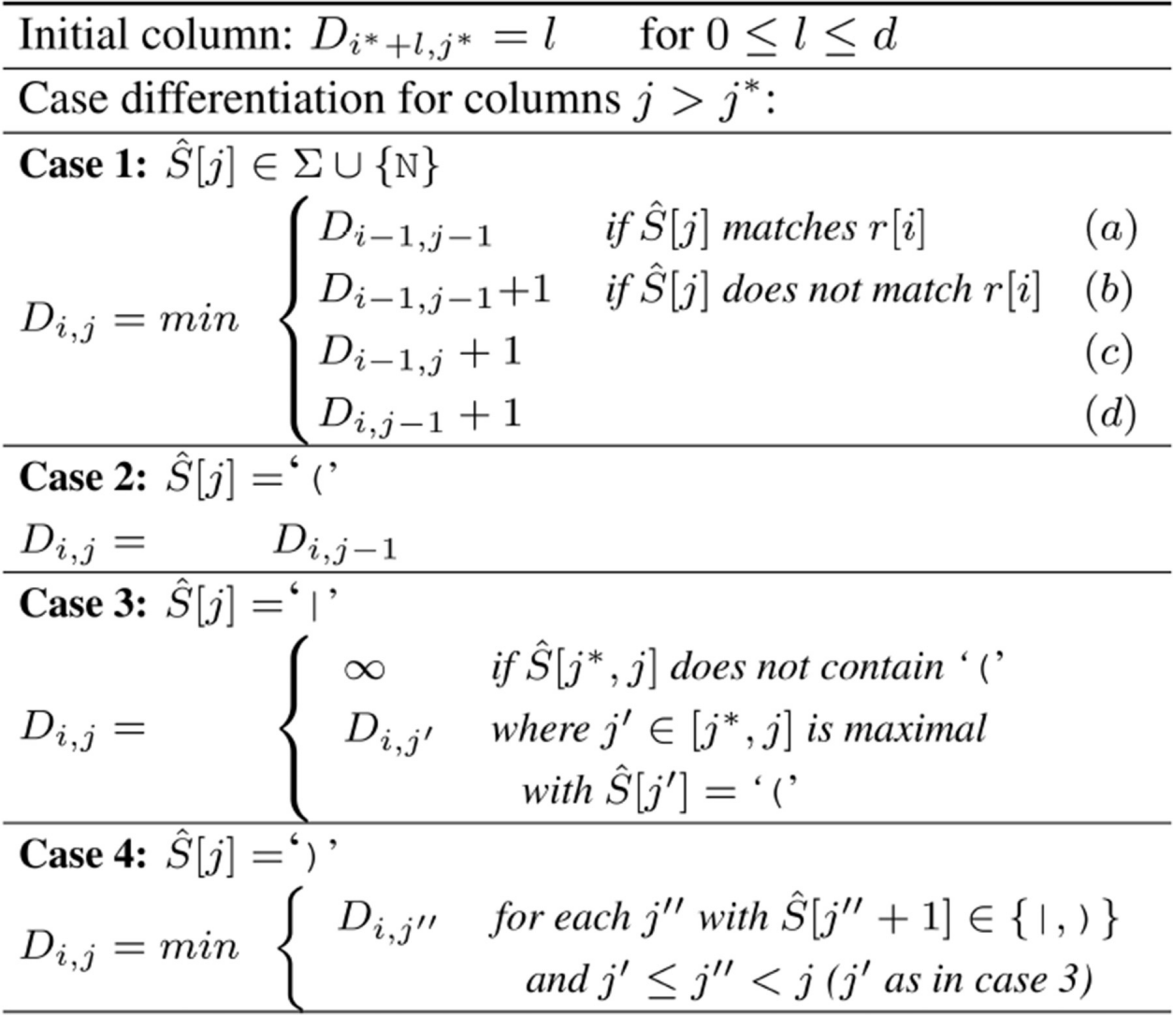

a The cases are: match (1a), mismatch (1b), insertion (1c), deletion (1d); start of an ED symbol (2); start of another alternative (3); end of an ED symbol (4).

**Table 3.** Alignment table *D* for $\hat{G}[11,22]=$A(AT|TA)GA#T, r[5,10]=AATCGA, and maximum distance 3.^a^

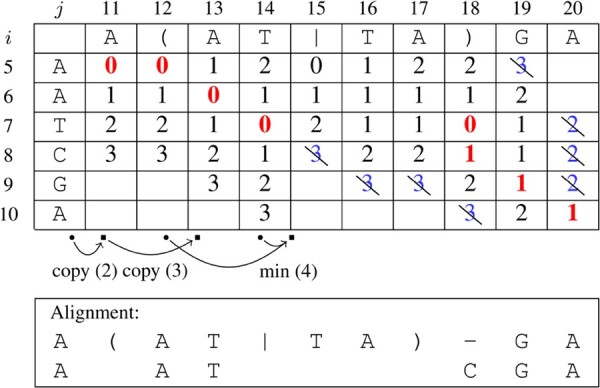

a Arrows beneath the table illustrate the special cases (2–4). Bold red numbers indicate the path of the best alignment. Crossed out blue numbers will not be calculated, since a complete alignment with better or equal distance was already observed. The alignment itself is shown below the alignment table.

We calculate the table column-wise and for each column *j* we only calculate the rows *i* that can have values ≤d and less than the distance of the currently best semi-global alignment if one has been found already. For example, in column j=14, we detect a semi-global alignment with 3 errors. Hence, we can ignore all values greater than or equal to 3 in the following columns. They are blue in Table 3 and will not be stored. Furthermore, in column 20 we detect a semi-global alignment with one error. Since there is no smaller value in this column, we can stop because there is no better alignment.

The alignment of *r*[1, 5] with $\hat{G}$ in backwards direction, which completes the example above, is given in the Supplementary Material. This alignment includes the insertion and spans over the node boundary “#” in the graph.

## 3 Experiments

To evaluate our software GED-MAP, we built an index of the human genome and its variants and tested the mapping of sampled and real reads. We compared our approach with VG-Giraffe (Garrison et al. 2018, Sirén et al. 2021) and HISAT2 (Kim et al. 2019). Additionally, we ran the same experiments with Minimap2 (Li 2018). Minimap2 works on a linear reference and does not include variations. Its index stores the minimizers of the reference sequence (similar to our approach).

Time and space consumption was measured externally with the terminal program “time.” Time refers to wall clock and space to the maximum resident set size. The server used to perform the experiments runs Ubuntu 20.04 (64 bit) with an AMD EPYC 7742 64-core processor, 256 GB RAM and 500 GB swap space on an SSD. For mapping, each program used 8 threads. Sources of our tool and scripts to replicate the experiments are available at github.com/thomas-buechler-ulm/gedmap. A short overview of the program calls and parameters can be found in the Supplementary Material.

### 3.1 Data

We used the GRCh37 reference and the variants of the 1000 Genomes Project (1000 Genomes Project Consortium 2015). This sequence consists of over 3 billion bases and includes over 84 million variants. Over 81 million variants are SNPs, about 3.3 million small indels and alternatives, and 44.9k are structural variants (42.6k copy number variations and 2.3k large indels). (There are less than 17k entries in the VCF file that describe insertions of mobile elements and inversions. These entries are currently neither supported by our software nor by the software we are comparing with.)

We used two different sets of real reads. The first set (ftp.sra.ebi.ac.uk/vol1/fastq/SRR769/SRR769545/SRR769545_2.fastq.gz) contains over 30 million 100 bp reads of a British male and was published by the International Genome Sample Resource. The reads in the second set (ftp-trace.ncbi.nih.gov/ReferenceSamples/giab/data/NA12878/Garvan_NA12878_HG001_HiSeq_Exome/NIST7035_TAAGGCGA_L001_R1_001.fastq.gz) originate from the pilot genome NA12878 of the Genome in a Bottle project (Zook et al. 2016) and contains 20 million 100 bp reads. The sampled reads were generated as follows. First, take a substring of the pangenome at a position drawn uniformly at random. For loci with known variants, an alternative is selected uniformly at random. Then errors are added to this substring. An error may be a substitution, insertion or deletion of a base. The probability for each error is 0.1% per base. We generated three test sets, each containing 100 million samples. The first and second set are containing 100 and 250 bp samples, respectively. To evaluate if reads containing structural variations can be mapped, the third set solely contains 150 bp samples that span over edges in the EDS graph $\hat{G}$.

### 3.2 Index generation and comparison

We use (5,20)-minimizers for our index. When choosing values for *w* and *k*, there is a trade-off between mapping speed, index size, and accuracy. In general, larger values for *k* increase the mapping speed and index size. Larger values for *w* are also increasing the mapping speed but are decreasing the accuracy. An evaluation of the performance of the minimizer index for different values of *w* and *k* can be found in the Supplementary Material. Moreover, experimental results reported there show that in virtually all cases there are enough matching (5,20)-minimizers between a read and an EDS to place the read correctly.

Table 4 shows the resources required by the tools to build the whole human pangenome index. Among the pangenomic tools, GED-MAP is the fastest and most space-efficient in building its index. Since HISAT2 needed more than 250 GB RAM, it had to make use of the swap space on SSD. Clearly, this considerably slowed down the construction of the index.

**Table 4.** Comparison of the index sizes on disc and the resources needed to calculate the index.^a^

Program	Time	Memory usage (GB)	Index size (GB)
GED-MAP	10 min	58.5	9.8
VG	12 h	203.3	115.1
HISAT2	37 h	650.0	16.1
Minimap2	1 min	11.4	8.5

a The row “VG” refers to the generation of all indexes needed to run VG-Giraffe. HISAT2 first transforms the VCF file into its own format. The time for this transformation is not included.

The index size is the space of all data needed for mapping. For GED-MAP this is 5.9 GB for the minimizer index and 3.9 GB for the EDS graph.

### 3.3 Comparison of mapping speed and accuracy

We mapped the reads to the pangenome and the linear reference, respectively, with the above-mentioned tools and evaluated the mapping rate, time, and space consumption.

**Figure 3.**
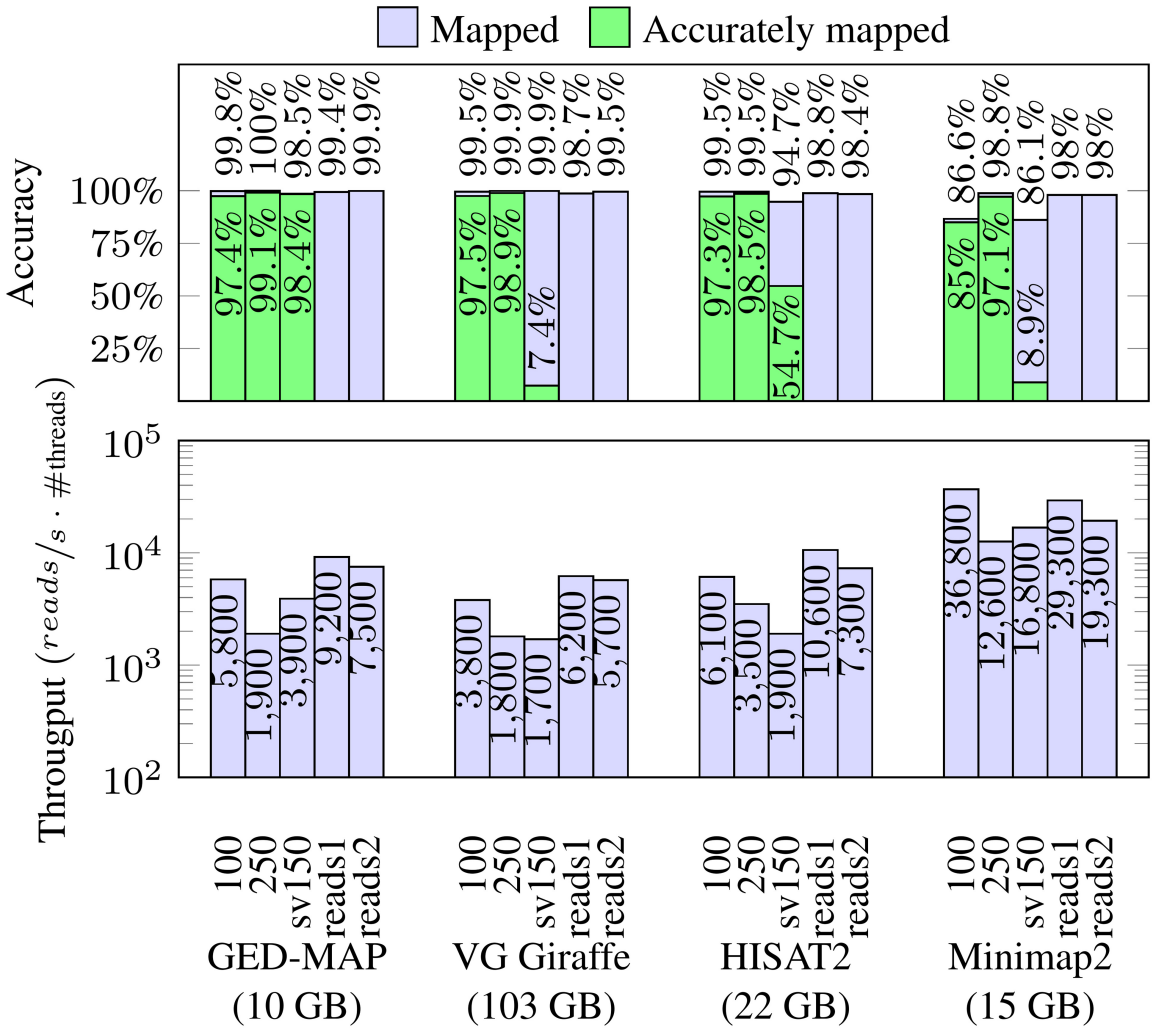
Evaluation of the accuracy, throughput, and space consumption of the mapping process. The upper chart is showing how many reads were aligned. For sampled reads, the proportion of correct alignments is additionally depicted (an alignment is correct if the sample was placed at the locus it was generated from). The percentages are rounded to one decimal place. The lower chart depicts the mapping speed in terms of number of reads per second and thread. All programs were run using eight threads. Please note the log scale of the lower plot. The observed space consumption is given in brackets below the chart.

We ran VG Giraffe with default parameters. HISAT2 was used with preset “sensitive” to achieve a similar accuracy as the other graph-based tools. Minimap2 was used with the preset for short reads. The results of these experiments are shown in Fig. 3. For all sets we show the mapping rate, i.e. the fraction of reads for which an alignment was generated. For the sets containing samples, we additionally depict the accuracy. We define an alignment reported by the mapping software as accurate, if its starting position equals (or is very close to) the position the sample was generated from.

For the 100 bp samples, the graph-based tools achieve a mapping rate in range of 99.5–99.8% with an accuracy in range of 97.3–97.5%. Note that Minimap2 is not competitive in this case. All tools are more accurate in mapping the 250 bp samples. Note that GED-MAP has the best accuracy of 99%.

Regarding the set of 150 bp samples representing structural variations, GED-MAP aligns 98.4% of the reads correctly. Since the other tools do not take copy number variations (CNVs) into account (recall that the included structural variations consist of 42.6k CNVs and 2.3k large indels), their mapping accuracy should be much lower in this case. Although CNVs are not included in the index of the other tools, their mapping accuracy is not as low as expected. This is because all tools apply clipping, i.e. only a partial alignment is computed (a substring of the read is mapped to a substring of the genome). Presumably, most of these partial alignments are part of the correct semi-global alignment. If the aligned part contains the front of the sequence, the reported position will be sufficiently close to the sampled position and counts as correct in our evaluation. (At the moment, GED-MAP does not use clipping.)

In terms of mapping speed, the linear mapper Minimap2 is 2–6 times faster than the pangenomic tools. HISAT2 and GED-MAP are up to two times faster than VG Giraffe. Furthermore, one can observe that each tool needs more time to place longer samples. GED-MAP is the most space efficient tool—even more efficient than the linear mapper. VG needs an order of magnitude more memory than the other tools. In all our evaluations, we just looked at the primary alignments. This means that when a tool finds more than one alignment for a read, only the best one is taken into account. Therefore, a correctly found alignment could be overlooked if the tool scores another alignment higher.

## 4 Discussion

In this article, we proposed a new graph representation for a pangenome. We build our graph from a reference sequence and a VCF file. Most variations, like SNPs and small indels, are encoded in place using a syntax well known from regular expressions. To our knowledge, the generation of the nodes and edges for copy number variations, is a new approach in mapping software.

The experiments show that the 150 bp samples representing structural variations can (partially) be mapped with the help of other techniques as well, but including these variations in the index improves the accuracy remarkably. The linearized form of our representation provides a natural and sensible coordinate system, which allows us to (a) use a minimizer index, (b) easily estimate distances, and (c) to cluster seed positions efficiently.

As a proof of concept, we developed the prototypical short read mapping software GED-MAP. We tested our software on sampled and real reads and compared it to VG Giraffe, HISAT2, and Minimap2. In our experiments, all graph-based tools achieved a similar mapping rate and accuracy. HISAT2 was slightly faster than GED-MAP, but its index construction is very slow and requires a lot of memory. GED-MAP was the most memory efficient tool. All tools provide parameters to control the mapping speed and rate. (A higher throughput comes with a lower mapping rate.)

Moreover, HISAT2 is merely able to incorporate variations that can be expressed by EDS. If one is interested in indexing structural variations, tools like VG-Giraffe and GED-MAP are recommended. The memory footage of GED-MAP is remarkably low compared to VG-Giraffe.

Unsurprisingly the graph-based tools were clearly slower than the linear mapping tool Minimap2. Since the kind of index Minimap2 uses is very similar to the index used by GED-MAP, it might be possible to further improve GED-MAP by implementing heuristics that are used in Minimap2. We are planning to evaluate this in the future. Additionally, we are aiming to implement features like clipping, paired end mapping, etc.

## Supplementary Material

btad320_Supplementary_Data

## Data Availability

The data underlying this article can be downloaded and generated using the instructions at https://github.com/thomas-buechler-ulm/gedmap#data.
