# Supplementary material for: Efficient short read mapping to a pangenome that is represented by a graph of ED strings
Source: Bioinformatics. 2023 May 12;39(5):btad320. doi: 10.1093/bioinformatics/btad320 (PMC10232250; doi:10.1093/bioinformatics/btad320)
Supplement: btad320_Supplementary_Data [file btad320_supplementary_data.pdf]

Supplementary material

The second part of the example from Section 2.5.2

In the example, the read r =TTAGAATCGA should be aligned with the EDS graph $\hat{G}$ = TT#NG(A|)CA(AT|TA)GA#T using $(5, 11)$ as seed and maximum 3 errors (see Section 2.5.2). The forward alignment of $r[5, 10]$ to $\hat{G}[11, 22]$ is already described in the main document (see Table 3). Since this already uses 1 error, we allow 2 errors in the alignment of $r[1, 5]$ to $\hat{G}[1, 11]$. In the following, we elaborate on the backward alignment of $r[1, 5]$ to $\hat{G}[1, 11]$. For better understanding, we show the alignment of the reverse strings in forward direction (see Figure 1) instead of depicting the alignment actually backwards. This means we align AGATT with C(|A)GN#... (the brackets '(' and ')' are swapped for better readability). Note: column 6 in the figure is the row wise minimum of columns 7 and 9. Furthermore, at column 3 the end of the node is reached ($\hat{G}[3]$ =#) . The possible predecessor positions are 3 and 21. We continue the alignment in two separate procedures at these positions. Only the procedure continuing at position 3 will yield an alignment with at most 2 errors. We trace back the alignment from (1,1) to (5,11).

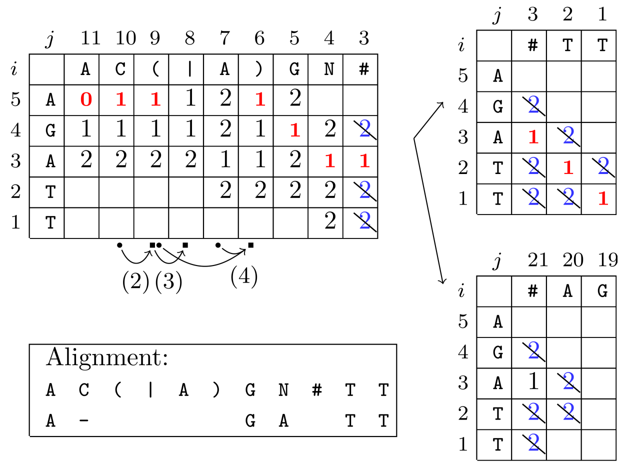

Fig. 1. Alignment table D for $\hat{G}[3, 11]$ = A(AT|TA)GA#, $\hat{G}[1, 3]$ = #TT, and $\hat{G}[19, 21]$ = #AG, $r[1, 5]$ = TTAGA, and maximum distance 2. Arrows beneath the table indicate the involved columns in the special cases (2-4). Bold red numbers indicate the path of a best alignment. At column 3, the alignment is continued at positions 3 and 21, because these are the positions of the preceding nodes.

Program calls in our experiments

In all program calls in this section, the common in- and output files are called FA, VCF, FQ and OUT.

GED-MAP

In the following, GED is the string of $\hat{G}$. GED.adj stores the adjacency list and GED.2fa is the bit-vector used to transform a coordinate in $\hat{G}$ to a coordinate in the reference. MINI is the minimizer index. The default parameters for the minimizer are $w = 5$ and $k = 20$. Likewise by default a trimming that removes all minimizers with more than 1000 occurences is applied. The flag 'rc' enables search for reverse complement and 'tc' sets the number of used threads. The parameters 'mac' and 'mat' are giving a limit to how often the dynamic programming algorithm will be started and executed completely. The parameter 'd' gives the maximum distance for the alignment. These parameters were set differently on different read sets.

```
gedmap parse FA VCF GED
gedmap index GED -o MINI
  -a GED.adj -2fa GED.eds2fa
gedmap align GED FQ MINI -o OUT
  -2fa GED.eds2fa -a GED.adj -rc -tc 8
  (-d 15/100 -mat 10/1000 )
```

HISAT2

We used version 2.2.1. The index was generated with default commands. EXT contains the extracted variation and IDX is the prefix for all index files. HISAT2 offers many parameters to customize the mapping process. With the chosen parameter setting, the internal scoring function equals the edit distance.

```
hisat2_extract_snps_haplotypes_VCF.py
  -non-rs FA VCF EXT
hisat2-build -f --large-index --snp EXT.snp
  --haplotype EXT.haplotype FA IDX
hisat2 -q -x IDX -U FQ -S OUT --threads 8
  --sensitive --no-softclip --np 0 -k 1
```

VG Giraffe

We used version v1.37.0-11 'Monchio' of the vg-toolkit. In the following commands, VG is the variation graph and XG is a more space-efficient representation of VG. GFA is the graph in GFA format. IDX is the prefix of the indexes used for the mapping process. These indexes include the GBWT, the corresponding GBWT graph, a minimizer index, and a distance index. We then mapped FQ to the index with default parameters and converted the output to SAM format.

```
tabix -p vcf VCF
vg construct -r  FA -v VCF.gz > VG
vg index -x XG VG
vg view VG > GFA
vg autoindex --workflow giraffe
  -g GFA -p IDX
vg giraffe -Z IDX -f FQ -t 64 -o SAM
  > OUT
```

Minimap2

We used verion 2.24 of Minimap2. In the following commands, IDX is the minimizer index.

```
minimap2 -x sr -d IDX FA
minimap2 -a IDX -t 8 FQ
```

Layout of the minimizer index

Let $\{\omega_1, \omega_2, \ldots, \omega_{(4^k)}\}$ be the set of k-mers over the alphabet $\{A, C, G, T\}$. We denote the k-mer with lexicographic rank j by ω_j. Further, let $M(\omega_j)$ be the set of positions at which ω_j occurs as minimizer in the EDS graph. For example in Figure 2 in the main document $M(\text{AGA}) = \{4, 17\}$ and $M(\text{AAA}) = \emptyset$. The purpose of the index is to determine $M(\omega_j)$ given a k-mer ω_j.

In the following let n be the number of k-mers w such that $M(\omega) \neq \emptyset$ and m be the number of k-mers with $|M(\omega)| = 1$. Our index layout is similar to SSHash [A] and also exploits the facts that for high enough values of k, firstly n is magnitudes smaller than 4^k (the index is sparse) and secondly m is not much smaller than n (non-empty sets $M(\omega)$ are often singleton).

We use a bit array *member* of size 4^k to indicate if a k-mer is present in the index, i.e. $member[j] = 1 \Leftrightarrow |M(\omega_j)| > 0$. This array is compressed using the Elias-Fano encoding. We further enhance *member* with additional data to support rank queries ($rank(member, x) := \sum_{i=1}^{x} member[i]$) in constant time. With rank queries we can map every k-mer, which is present in the index, to a value in $\{1, \dots, n\}$. We define $h : \{1, \dots, 4^k\} \rightarrow \{1, \dots, n\}$ with $h : j \mapsto rank(member, j)$). (So the function h corresponds to the Minimal Perfect Hash Function in [A]. Additionally *member* allows us to answer membership queries in constant time.)

We use a second bit array *singleton* of size n to indicate if the set $M(\omega)$ is singleton, i.e. $singleton[h(j)] = 1 \Leftrightarrow |M(\omega_j)| = 1$. The array *singleton* is also enhanced with a rank support.

We use two arrays pos_S and pos_M that contain the actual positions of the k-mers. (These vectors correspond to the vector *Offset* in [A].) The array pos_S contains the values of all sets $M(\omega)$ that are singleton, while pos_M contains the values of the sets $M(\omega)$ that are not singleton. If $M(\omega_j)$ is singleton, we can compute $M(\omega_j)=\{pos_S[x]\}$ with $x = rank(singleton, h(j))$.

To be able to report the elements of the non-singleton sets, we need an additional vector *start* (similar to *Sizes* in [A]. If ω_j is the y-th k-mer with $|M(\omega_j)| > 1$ then $pos_M[start[y]]$ stores the first value of $M(\omega_j)$. We can determine y by $h(j) - x$, where $x = rank(singleton, h(j))$. The set $M(\omega_j)$ is $\{pos_M[start[y]], \dots, pos_M[start[y+1]] - 1\}$. (To ensure that $start[y+1]$ is always defined, we set $start[n-m] = |pos_M| + 1$.)

For instance, the arrays for the index shown in Figure 2 of the main document are: $pos_S = [20, 11, 7, 13]$, $pos_M = [4, 17]$, $singleton = [1, 1, 1, 0, 1]$, $start = [1, 3]$ and *member* contains ones for AAG, AAT, ACA, AGA, and ATG. In this example, ATG is the fifth k-mer in the index and $singleton[5]$ is the fourth 1 in *singleton*. Therefore $M(\texttt{ATG}) = \{pos_S[4]\} = \{13\}$. Furthermore, AGA is the fourth k-mer and $singleton[4]$ is the first 0 in *singleton*. Hence we have $pos_M[start[1]..start[1+1]-1] = pos_M[1..2] \hat{=} \{4, 17\} = M(\texttt{AGA})$.

The index used for the experiments requires 5.9 GB storage. This devides in 3.4 GB for pos_S, 0.7 GB for pos_M, 1.5 GB for *member* and 0.2 GB for *start* and 0.1 GB for *singleton*.

[A] G.E. Pibiri, Sparse and skew hashing of K-mers, Bioinformatics, **38**, Supplement 1, pages i185–i194, 2022.

Evaluation of the performance of the minimizer index for different values of k and w

The two main parameters for the index are the length k of a k-mer and the window size w. If k or w is high, there are fewer positions per minimizer in the index. Furthermore, higher values of k or w are reducing the number of minimizers per pattern. This results in a low number of positions per pattern, which leads to a higher mapping speed. When the number of positions per pattern is low, however, the algorithm may not be able to place the pattern correctly and hence the accuracy is lower.

To evaluate this effect, we built the index for the whole human genome with different parameters of k and w and mapped 1 million generated samples using the index. We measured the following values:

- the average number of positions, loaded from the index, per sample
- the mapping speed
- the average number of ‘correct’ positions (positions that are part of the correct alignment), loaded from the index, per sample.
- the accuracy, i.e. the fraction of correctly placed samples
- the size of the index

As described in the main document, we applied a trimming, which removed all k-mers with more than 1000 occurrences from the index. Our evaluation is shown for the index before and after the trimming.

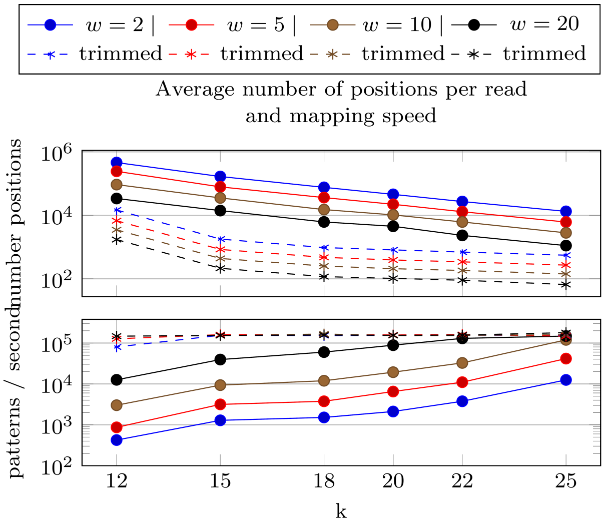

Fig. 2. The upper diagram is showing the average number of positions loaded from the index, when querying a sample. The lower diagram is the throughput of the mapping process in patterns per second. Both axes are semi logarithmic. The number of positions per read decreases exponentially, when k increases. The larger the window is, the fewer positions there are per pattern. The different colors indicate different window sizes. The dashed line is showing the values after the trimming is applied. One can observe that the mapping speed behaves inversely proportional to the average number of positions.

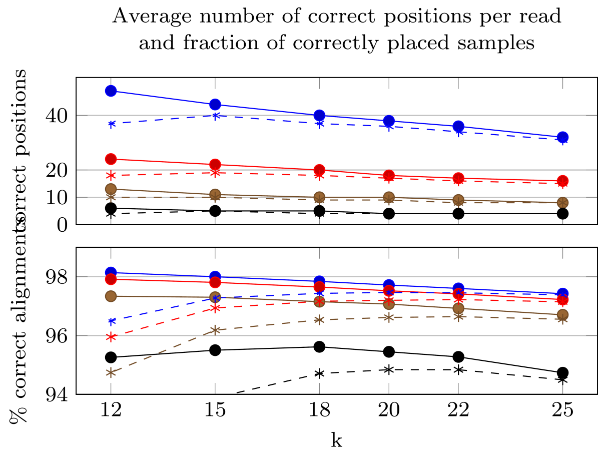

Fig. 3. The upper diagram is showing the average number of positions (per pattern), that are loaded from the index and lead to the correct alignment. The lower diagram is showing the fraction of samples that were aligned to the position they were generated from. When k or w increase, the number of 'correct positions' and the number of correctly placed samples decrease. Furthermore, applying the trimming on an index reduces its accuracy. This effect is minor, when $k \geq 18$ and $w \leq 10$. (Legend as in Figure 2.)

On the one hand, to be able to find the correct alignment, we would like to have a high number of ‘correct’ positions. On the other hand, to be fast, we would like to have a low number of positions per read. Figure 2 is showing that the number of positions per read decreases exponentially (note the semi logarithmic scale), when increasing k or w. The throughput behaves inversely proportional to the number of positions until it reaches a limit approximately at 2 - 3 $\times 10^5$ patterns per second. To be able to place the pattern correctly, the number of ‘correct’ positions is more relevant. This is shown in Figure 3 along with the accuracy of the mapping process. One can see that the number of ‘correct’ positions decreases slowly as k increases. Furthermore, we see that the trimming remarkably lowers the

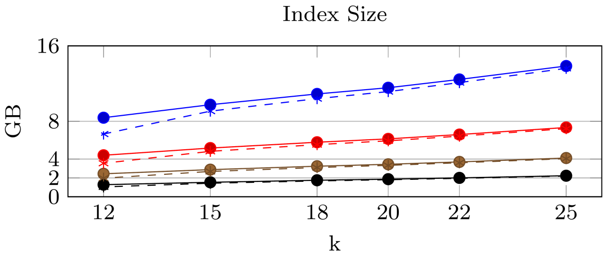

Fig. 4. The index size in GB. (Legend as in Figure 2.)

number of positions per read while having a minor influence (at least for $k > 12$) on the number of 'correct' positions. The limit of the accuracy is around 98%. Lower values of w lead to a higher accuracy.

The index size is shown in Figure 4. A base line for the index size is the space needed to store the positions (in the arrays pos_S and pos_M). That strongly depends on w and is quite independent of k. An increase of k mainly increases the space needed to store the array $member$.

For a good choice of the values for k and w as well as the trimming, one has to consider a space-time-accuracy trade off. A higher space consumption comes with a higher accuracy and a lower mapping speed.

For our experiments we choose $w = 20$ and $k = 5$. With these values the minimizer index has a size of 5.9 GB and stores positions for over 879 million k-mers. That is about 6.7 bytes per k-mer. (We already need 4 bytes to store a single position of a k-mer.) The trimming removed about $1.5 \cdot 10^4$ k-mers with a total of over 51 million positions.

Applying a non lexicographic hash function counters the loss of alternative alleles

At a fixed position in the EDS graph, different k-mers may start if there is a branch right behind this position. Instead of considering all k-mers of such a position in the minimizer index, we focus on the k-mer with the minimum hash value. Hence the k-mer at this position does only represent one alternative.

When using the lexicographic rank as the hash value of a k-mer (as done in the examples of this article), all k-mers, which start right before a branch, will represent the lexicographic smallest alternative and so the lexicographic larger alternatives will not be covered properly; see Figure 5. There are three minimizers that cover the variant `(G|T)` and all of them cover the alternative `G`. Hence the alternative `T` is not present in the index.

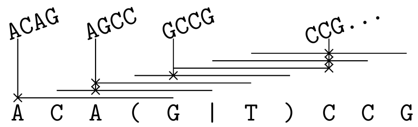

Fig. 5. Lexicographic minimizer for $k = 4$ and $w = 5$ of the ED string `ACA(G|T)CCG`. A vertical line above the string represents a windows. The cross on each line marks the minimizer of this window.

This effect can be countered by applying a more random-like hash function, as done in the actual implementation. If the hash values behave like random values, an alternative of a variant will be covered with each minimizer that overlaps this variant. Suppose the hash function maps the k-mers in the example to their lexicographic rank, except `ATCC` is mapped to a lower hash value than `AGCC`. Then the second minimizer would be `ATCC` instead of `AGCC` and both alternatives are covered in the index.

This does not guarantee that all alleles are present in the index. Nevertheless, our experiments show that, with a suitable choice of parameters, there are in practice enough matching minimizer to be able to place a pattern correctly. As one can see in Figure 3, there are on average almost 20 matching minimizers between a pattern and the EDS with the parameters of our experiments.

Comparison of the graph based tools on the linear reference

We repeated the experiments of Section 3.3 on the linear reference. That is, we built the indexes for GED-MAP, VG-Giraffe and HISAT2 without the variant-information (Table 1) and then executed the mapping on the same input as in the original experiments (Figure 6). The most remarkable observation in Table 1 is that, without variations, the index construction of HISAT2 is fast and memory efficient.

Table 1. Comparison of the index sizes on disc and the resources needed to calculate the linear index. The row 'VG' refers to the generation of all indexes needed to run Giraffe.

program	time		memory usage (GB)	index size (GB)
GED-MAP	7	min	52.7	8.7
VG	3	h	135.9	41.1
HISAT2	14	min	17.4	5.1
Minimap2	1	min	11.4	8.5

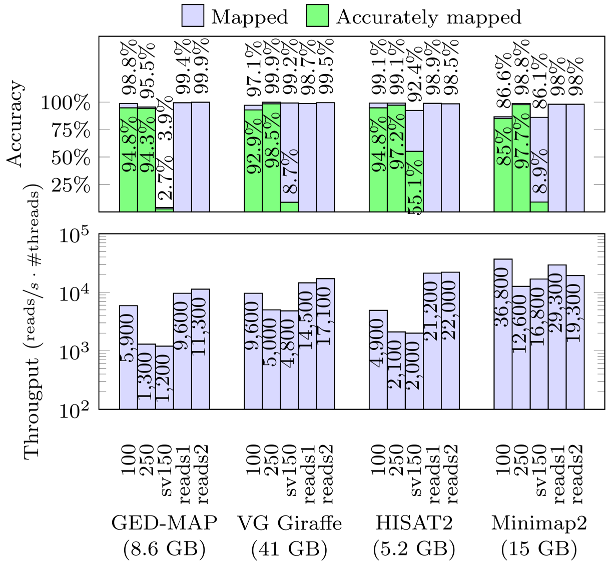

Fig. 6. Evaluation of the accuracy, throughput, and space consumption of the mapping process. This figure shows the same evaluation as Figure 3 in the main document, but without using variants in the graph based tools.
